# Comparison of the Bone Harvesting Capacity of an Intraoral Bone Harvesting Device and Three Different Implant Drills

**DOI:** 10.1155/2017/7819080

**Published:** 2017-12-14

**Authors:** Hyun-Chang Lim, Kyung-In Ha, Ji-Youn Hong, Ji-Young Han, Seung-Il Shin, Seung-Yun Shin, Yeek Herr, Jong-Hyuk Chung

**Affiliations:** ^1^Department of Periodontology, Periodontal-Implant Clinical Research Institute, School of Dentistry, Kyung Hee University, Seoul, Republic of Korea; ^2^Department of Dentistry/Periodontology, College of Medicine, Hanyang University, Seoul, Republic of Korea

## Abstract

The aim of the present study was to compare bone-collecting capacity of bone harvesting device and minimally irrigated low-speed drilling using three implant systems. One bone harvesting device and three commercially available drill systems were compared using the osteotomies on bovine rib bones. The amount of the collected bone particle and particle size (<500 *μ*m: small, 500–1000 *μ*m: medium, and >1000 *μ*m: large) were measured. Total wet (1.535 ± 0.232 mL) and dry volume (1.147 ± 0.425 mL) of the bone particles from bone harvesting device were significantly greater than three drill systems (wet volume: 1.225 ± 0.187–1.27 ± 0.29 mL and dry volume: 0.688 ± 0.163–0.74 ± 0.311 mL) (*P* < 0.05). In all groups, the amount of large sized particles in wet and dry state was the greatest compared to that of medium and small particles. The dry weight of the bone particles showed the same tendency to volumetric measurement. In conclusion, total bone particles and large sized particles (>1000 *μ*m) were harvested significantly greater by bone harvesting device than minimally irrigated low-speed drilling. The composition of particle size in all harvesting methods was similar to each other.

## 1. Introduction

Autogenous bone is considered the gold standard for implantation because of its biocompatibility and osteogenicity [1, 2]. Peri-implant defects, such as dehiscence or fenestration, have been successfully managed by grafting autogenous bone [3–5]. Autogenous bone for intraoral augmentation can be collected in either block or particulate form and can be used alone or in combination with other bone substitutes from different origins [5–11].

Harvesting autogenous bone is generally presumed to involve a second surgical site and higher patient morbidity. However, autogenous bone can be collected during preparation for implant osteotomy, which is highly advantageous for the patient because it avoids the above-mentioned limitations [12]. The main clinical challenge in collecting bone particles during implant drilling is irrigation. Without proper cooling by irrigation, overheating and subsequent alveolar bone necrosis can occur. An aspiration technique using a specially designed suction device has been proposed, but this may render the collected bone unusable due to contamination by oral bacteria and debris [13]. More recently, a minimally irrigated low-speed drilling technique has been used to collect a significant amount of bone particles [14, 15]. This technique is relatively convenient and easy compared to other autogenous bone harvesting techniques, both device-wise and time-wise.

Commercially available bone substitutes are provided at a uniform range of particle sizes, but the minimally irrigated low-speed drilling technique appears to result in various particle sizes. Bone-forming potential could vary with bone particle size [16]. However, there have been few studies exploring the size of harvested particles from drilling or bone harvesting device. Moreover, the quantity of bone particles that can be obtained using various techniques should be investigated because different peri-implant defects require different amounts of bone substitute.

The aim of the present study was to compare the bone harvesting capacity, that is, the size and amount of harvested bone particles, of a specially designed bone harvesting device and minimally irrigated low-speed drilling using three implant systems.

## 2. Materials and Methods

### 2.1. Experimental Model

The experiment protocol was based on previous studies with modifications [17, 18]. Briefly, bone harvesting for collection of bone particles was performed on bovine rib bones. Bones with >10 mm wide surface were selected. Periosteum was removed to expose the cortical bone. The cortical bone thickness of the bone was measured with a digital caliper (Digimatic caliper, Mitutoyo Co., Kawasaki, Japan).

### 2.2. Bone Harvesting Device and Drill Systems

**Figure 1:**
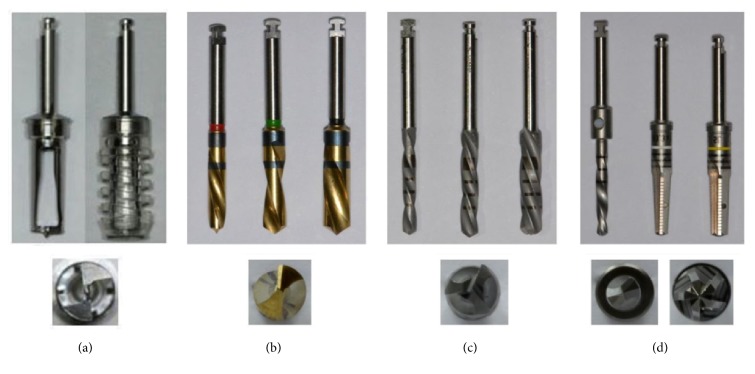
Devices used in the experiment, (a) Group 1. Bone harvesting device, (b) Group 2. Three two-flute sequential twist drills (Ø 2.5, 3.2, 3.7 × 11 mm), (c) Group 3. Three three-flute sequential twist drills (Ø 2.2, 2.8, 3.5 × 10 mm), (d) Group 4. One two-flute pilot drill (Ø  2.0 × 11 mm) and two five-flute sequential drills (Ø 3.3, 3.8 × 11 mm).

One bone harvesting device (Group 1) and drills from three different implant systems (Groups 2, 3, and 4) were used for bone collection. The device for Group 1 includes a cylindrical trephine-like drill (Ø: 4 mm) with a hollow part in the center and a stopper which limits the depth of drill penetration up to 4 mm (Neo AutoChip Maker, Neobiotech, Seoul, Korea). The drills for Groups 2, 3, and 4 have different numbers of flutes. For Group 2, three two-flute parallel-shaped twist drills were sequentially used (Ø 2.5, Ø 3.2, and Ø 3.7 × 11 mm; Astra Tech Implant System, Mölndal, Sweden). For Group 3, three three-flute parallel-shaped twist drills were sequentially used (Ø 2.2, Ø 2.8, and Ø 3.5 × 10 mm; Straumann, Basel, Swiss). For Group 4, one two-flute parallel-shaped twist drill (Ø 2.0 × 11 mm; Camlog, Basel, Swiss) and then two tapered-shaped five-flute straight drills (Ø 3.3 and Ø 3.8 × 11 mm; Camlog) were sequentially used (Table 1, Figure 1).

### 2.3. Bone Harvesting and Measurement

Ten osteotomies in one rib bone were performed for each group (10 rib bone for each group) using a Surgic XT plus (NSK, Kanuma, Japan). Drilling speed and torque were set at 100 rpm and 30 Ncm, respectively. Osteotomy preparation was designed for installation implants with regular platform (around Ø 4.0 mm). Drilling was performed using the above-listed drills from smallest diameter to greatest diameter according to the manufacturers' instructions. Saline irrigation during the drilling was minimized according to a previous study [12]. The distance between drilling sites was 8 mm. Bone-collecting and measuring methods were described in our previous study [19]. Briefly, bone particles in the hollow part of the bone harvesting device and the flutes of the drills were collected by scraping into a small bowl. Remnant particles in the osteotomy holes were also collected by thorough saline irrigation. The collected bone particles were packed into a 2 mL syringe for removing excessive saline and measured total wet volume (JW-1 electronic-scale; Acom, Pochun, Korea). Then bone particles were sieved serially using two sieves (500 *μ*m and 1000 *μ*m; Chunggye Co., Seoul, Korea). The bone particles were divided into three categories based on particle sizes; <500 *μ*m (small particles; SPs), 500–1000 *μ*m (medium particles; MPs), and >1000 *μ*m (large particles; LPs). Then, the wet volume of each category was measured. After drying for 72 hours at room temperature, the total and categorical dry volumes were also measured.

### 2.4. Statistics

One rib bone was regarded as one unit for statistical analysis, and thus each group was composed of 10 units, respectively. The average value from each unit was served as a representative for the unit. Statistical analysis was performed using statistical software (SPSS ver. 18.0, SPSS Inc., Chicago, IL, USA). Data were presented as the mean and standard deviation (SD). The Kruskal-Wallis test, followed by the Mann–Whitney test, was used to identify significant differences. Statistical significance was set at *P* < 0.05.

## 3. Results

The cortical thickness of the bovine rib bone was 2.297 ± 0.051 mm on average.

**Figure 2:**
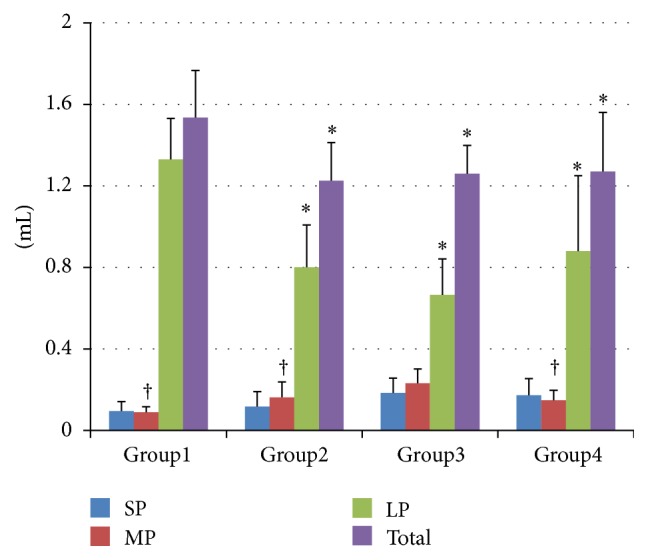
Wet volume (mL) of the bone particles (mean ± SD) collected during the 10 osteotomies. SPs: small particles, <500 *μ*m, MPs: medium particles, 500–1000 *μ*m, and LPs: large particles, >1000 *μ*m. ^*∗*^Statistically significant compared to Group 1 in the same category of bone particles. ^†^Statistically significant compared to Group 3 in the same category of bone particles.

The wet volume of bone particles collected during the 10 osteotomies (1 unit) in each of the four groups is shown in Figure 2. The total wet volume of Group 1 (1.535 ± 0.232 mL) was significantly greater than that of the other groups (Group 2: 1.225 ± 0.187 mL, Group 3: 1.26 ± 0.139 mL, and Group 4: 1.27 ± 0.29 mL, *P* < 0.05 for all comparisons). In intragroup comparisons of wet volume, particle size distribution in all groups showed a similar pattern; the amount of large particles (LPs, >1000 *μ*m) was greater than medium particles (MPs, 500–1000 *μ*m) or small particles (SPs, <500 *μ*m), while the amounts of MPs and SPs were similar. The wet volume of LPs in Group 1 (1.367 ± 0.175 mL) was significantly greater than that in other groups (*P* < 0.001 for Groups 2 and 3, *P* < 0.05 for Group 4). The wet volume of MPs in Group 3 (0.232 ± 0.069 mL) was significantly greater than that in Groups 1 (*P* < 0.001), 2 (*P* < 0.05), and 4 (*P* < 0.05).

**Figure 3:**
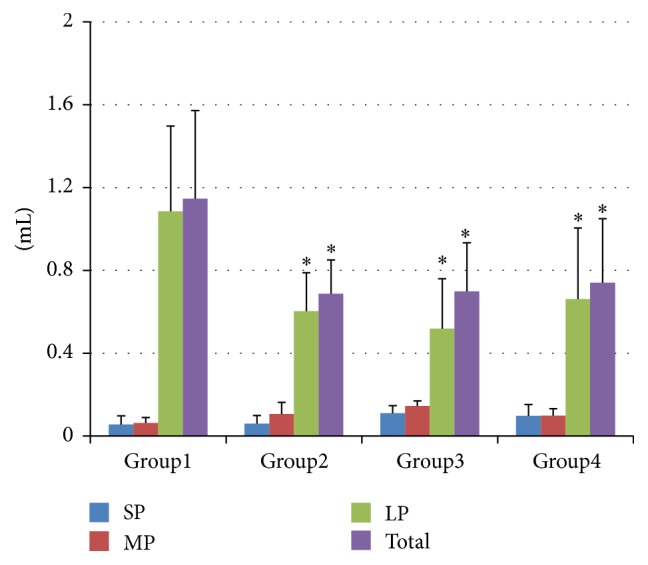
Dry volume (mL) of the bone particles (mean ± SD) collected during the 10 osteotomies. SPs: small particles, <500 *μ*m, MPs: medium particles, 500–1000 *μ*m, and LPs: large particles, >1000 *μ*m. ^*∗*^Statistically significant compared to Group 1 in the same category of bone particles.

The dry volume of bone particles is presented in Figure 3. The dry volume of particles in all groups was less than the wet volume. The total dry and LP volume of Group 1 (1.147 ± 0.425 mL) was significantly greater than in other groups (Group 2: 0.688 ± 0.163 mL, Group 3: 0.699 ± 0.235 mL, and Group 4: 0.74 ± 0.311 mL, *P* < 0.05 for all comparisons)

**Figure 4:**
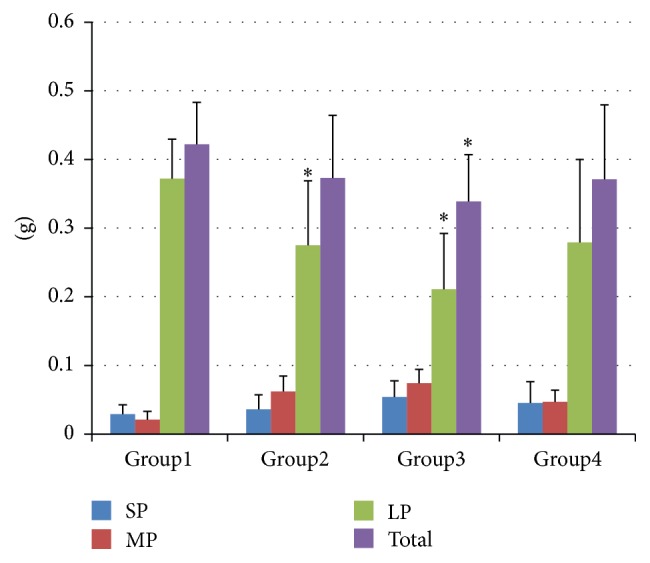
Dry weight (g) of the bone particles (mean ± SD) collected during the 10 osteotomies. SPs: small particles, <500 *μ*m, MPs: medium particles, 500–1000 *μ*m, and LPs: large particles, >1000 *μ*m. ^*∗*^Statistically significant compared to Group 1 in the same category of bone particles.

The dry weight of bone particles is presented in Figure 4. In all groups, more than 0.3 g of bone particles were collected in this study. The total dry weight of Group 1 (0.422 ± 0.061 g) was significantly greater than that of Group 3 (*P* < 0.05). The dry weight of LPs in Group 1 (0.372 ± 0.058 g) was significantly greater than that of Groups 2 (*P* < 0.001) and 3 (*P* < 0.05).

## 4. Discussion

In the present study, three commercially available drill systems were compared with a specially designed bone harvesting device. Bone collection via drilling is more convenient for the clinician and more patient-friendly compared to the use of a bone harvesting device. Irrigation to prevent excessive heat generation has been considered a major obstacle to harvesting autogenous bone via drilling because it flushes away bone particles. However, there is growing evidence that irrigation can be minimized during osteotomy preparation. Anitua et al. proposed novel low-speed (20–80 rpm) drilling procedure without irrigation [20]. The bone particles obtained by this procedure included well-preserved trabeculae in which osteocytes, osteoblasts, and lining cells could be found. Thermal imaging analysis revealed that low-speed drilling (50 rpm) without irrigation did not cause overheating [21]. Park et al. extracted alveolar bone-derived stromal cells from bone particles obtained via minimally irrigated dental implant drilling at low speed (50–200 rpm) and demonstrated that the cells have the potential to differentiate into osteoblasts and adipocytes [14]. To date, no studies have evaluated whether drills designed for osteotomy preparation have a similar capacity of bone harvesting to specially designed bone harvesting devices. The profile of the bone particles obtained using each drilling system, such as the amount obtained and particle size, could help confirm the clinical usefulness of these techniques.

The amount of harvested bone is dependent on the cortical thickness of the donor site. Previously, the cortical bone thickness of the human mandible was investigated. In a study using cone-beam computed tomography (CBCT), the mean thickness of buccal cortical bone was 2.5 mm and 3.18 mm at the apex of mandibular first and second molar, respectively [22]. In a cadaver study, the average buccal cortical plate thickness of the edentulous posterior mandible was 2.26–2.88 mm [23]. In the present study, the mean cortical bone thickness of the bovine rib bones was approximately 2.3 mm, which is clinically similar to the human mandible. At a single osteotomy site in the present study, the mean wet volume of harvested bone was 0.123–0.154 mL. In another ex vivo study, approximately 0.5 mL of bone particles was collected, but the model used was the bovine mandibular body (cortical bone thickness > 5 mm) [19]. Clinically, Savant et al. collected 0.195 mL of bone particles [24], and Kainulainen et al. collected 0.09–0.12 mL bone from a single implant site preparation in a human [25]. Considering this, only small peri-implant defects may be managed with the sole use of the autogenous bone harvested from a single osteotomy site, while a medium or large defect may require additional bone substitutes. Although the amount of the collected autogenous bone was not enough for sufficient contour around the implant, the autogenous bone can provide osteopromotive effect and the insufficient volume can be compensated with other bone substitutes [26, 27]. This type of augmentation, so called contour augmentation, has been documented well previously, applying autogenous bone chips on the denuded implant surface, covering slowly resorptive bone substitute, such as deproteinized bovine bone mineral, and covering the augmentation with collagen membrane [28].

Particle size may affect bone-forming capability and volume stability. Small particles increase surface area and subsequently increase the release of growth and differentiation factors [29], whereas they tend to be rapidly resorbed and hamper space maintenance [16]. Some studies demonstrated that particle size in the range of 250–1000 *μ*m is suitable for bone formation [30–33]. Shapoff et al. reported that bone particles ranging from 125 to 1000 *μ*m had greater osteogenic activity than particles <125 *μ*m [30]. Urist et al. reported that decalcified freeze-dried bone allografts ranging from 250 to 420 *μ*m resulted in better bone induction than those ranging from 1000–2000 *μ*m [31]. However, in other studies, LPs (1000–2000 *μ*m) led to significantly greater bone volume and vital new bone growth compared to SPs (150–400 *μ*m/250–1000 *μ*m) [34, 35]. Even though LPs (1000–2000 *μ*m) resulted in significantly less bone formation in the early healing period compared to SPs (250–1000 *μ*m), this difference disappeared over time [36]. In the present study, LP fractions (>1000 *μ*m) were harvested in greater amounts than MP and SP fractions. Despite the contradictory results of previous studies, the harvested bone from both the bone harvesting device and the drills used in the present study could be used for new bone formation without affecting volume stability.

When harvesting bone particles during osteotomy preparation, drill geometry may be important. Indeed, Park et al. suggested that drill morphology significantly influenced the size of collected bone particles [18]. However, there have been few studies on drill geometry for bone harvesting, and in some cases drill geometry is proprietary information of the implant company. In the authors' opinion, the number of flutes and web size should be emphasized for the total amount obtainable. The flutes are designed for discharging bone debris. A higher number of flutes indicate a narrower individual flute width, which is disadvantageous for trapping bone chips. Moreover, more flutes lead to a higher temperature during osteotomy preparation [37], which may increase the need for irrigation. The web is the core of the drill body that joins the blades. A narrower web results in a wider flute, which is beneficial for collecting bone chips, while the web dimensions should provide adequate drill strength. Other characteristics, such as helix angle, may also affect bone harvesting and should be further investigated.

The findings of the present study should be carefully utilized in clinical settings. First of all, clinicians cannot precisely predict cortical bone thickness from routine panoramic radiography. On three-dimensional radiography such as CBCT, the thickness can be measured preoperatively. If thin cortical bone is observed in CBCT, harvest of autogenous bone via drilling is unrealistic, and a bone harvesting device may be a better option considering that the total volume of bone harvested by the device was significantly greater compared to drilling. However, the device should be used several times at other surgical sites to obtain a sufficient amount.

Secondly, harvesting via drilling may not be feasible in bone that is too hard or soft. Osteotomy in hard bone requires high-speed drilling under a copious cooling system, in which case minimally irrigated low-speed drilling is out of the question. Soft bone sometimes requires undersized preparation, that is, reduced use of drilling, which decreases the amount of bone harvested. Also, soft bone provides an insufficient amount for harvest due to low density.

Collectively, the wet and dry volume of total bone particles and LP fractions were significantly greater when a bone harvesting device was used compared to drilling. The amount of autogenous bone harvested via drilling may be suitable for treating small peri-implant defects when it is used solely. However, the autogenous bone can be also usefully utilized due to its osteopromotive effect when it is used concomitantly with other commercial bone substitutes [26], although the amount of the harvested autogenous bone is small.

## 5. Conclusion

In the present study, a specially designed bone harvesting device and three different dental implant system drills were compared in terms of the amount of harvested bone and particle size. The amount of total bone particles and large particles (>1000 *μ*m) harvested was significantly greater when a bone harvesting device was used compared to minimally irrigated low-speed drilling with commercially available drills, but the particle size composition was similar.

## Figures and Tables

**Table 1:** Characteristics of each drill group.

Group	Drill shape	Flute number	Diameter (mm)	Length (mm)
1	Straight	2	4.0	14 (stopper 4 mm)

2	Parallel, twist	2	2.5	11
	Parallel, twist	2	3.2	11
	Parallel, twist	2	3.7	11

3	Parallel, twist	3	2.2	10
	Parallel, twist	3	2.8	10
	Parallel, twist	3	3.5	10

4	Parallel, twist	2	2	11
	Tapered, straight	5	3.3	11
	Tapered, straight	5	3.8	11
